# From Maternal Diet to Neurodevelopmental Disorders: A Story of Neuroinflammation

**DOI:** 10.3389/fncel.2020.612705

**Published:** 2021-01-15

**Authors:** Maude Bordeleau, Lourdes Fernández de Cossío, M. Mallar Chakravarty, Marie-Ève Tremblay

**Affiliations:** ^1^Integrated Program in Neuroscience, McGill University, Montréal, QC, Canada; ^2^Axe Neurosciences, Centre de Recherche du CHU de Québec-Université Laval, Québec, QC, Canada; ^3^Department of Neurosciences, University of California, San Diego, La Jolla, CA, United States; ^4^Cerebral Imaging Centre, Douglas Mental Health University, McGill University, Montréal, QC, Canada; ^5^Department of Psychiatry, McGill University, Montréal, QC, Canada; ^6^Department of Biological and Biomedical Engineering, McGill University, Montréal, QC, Canada; ^7^Département de Médecine Moléculaire, Université Laval, Québec, QC, Canada; ^8^Department of Neurology and Neurosurgery, McGill University, Montréal, QC, Canada; ^9^Division of Medical Sciences, University of Victoria, Victoria, BC, Canada; ^10^Biochemistry and Molecular Biology, Faculty of Medicine, The University of British Columbia, Vancouver, BC, Canada

**Keywords:** maternal diet, nutrient imbalance, inflammation, genetic programming, microglia, gut microbiome, neurodevelopmental disorders, schizophrenia

## Abstract

Providing the appropriate quantity and quality of food needed for both the mother’s well-being and the healthy development of the offspring is crucial during pregnancy. However, the macro- and micronutrient intake also impacts the body’s regulatory supersystems of the mother, such as the immune, endocrine, and nervous systems, which ultimately influence the overall development of the offspring. Of particular importance is the association between unhealthy maternal diet and neurodevelopmental disorders in the offspring. Epidemiological studies have linked neurodevelopmental disorders like autism spectrum disorders, attention-deficit-hyperactivity disorder, and schizophrenia, to maternal immune activation (MIA) during gestation. While the deleterious consequences of diet-induced MIA on offspring neurodevelopment are increasingly revealed, neuroinflammation is emerging as a key underlying mechanism. In this review, we compile the evidence available on how the mother and offspring are both impacted by maternal dietary imbalance. We specifically explore the various inflammatory and anti-inflammatory effects of dietary components and discuss how changes in inflammatory status can prime the offspring brain development toward neurodevelopmental disorders. Lastly, we discuss research evidence on the mechanisms that sustain the relationship between maternal dietary imbalance and offspring brain development, involving altered neuroinflammatory status in the offspring, as well as genetic to cellular programming notably of microglia, and the evidence that the gut microbiome may act as a key mediator.

## Introduction

Nutrition is of course essential to the maintenance of life, but it is particularly fundamental at the onset of life during the antenatal and early life periods of growth and development of organs and systems. Although diet holds great importance for proper development, macronutrients (carbohydrates, proteins, fats) and micronutrients (vitamins, minerals) are often consumed by pregnant and/or lactating mothers in inadequate proportions (Garcia−Casal et al., 2018). Across the world, ∼39 million pregnant women are estimated to be obese [body mass index (BMI) over 30] or overweight (BMI: between 25 and 30) due to poor nutrition (Chen et al., 2018), ∼32 million of pregnant women are anemic, ∼19 million of pregnant women suffer from vitamin A deficiency, while millions of pregnant women suffer from iron, folate, zinc, and/or iodine intake deficiency (Garcia−Casal et al., 2018). Malnutrition includes maternal undernutrition and nutrient deficiency but also excess of some key nutrients, like carbohydrates and fats, that often lead to maternal overweight and obesity (World Health Organization [WHO], 2020).

It is not surprising that maternal diet can profoundly impact fetal and early postnatal development of a mother’s progeny (Garcia−Casal et al., 2018). Indeed, malnutrition during *in utero* and early life, notably due to inappropriate quantity and quality of nutrients consumed by the mothers, can affect the offspring’s growth, metabolism, immune function, brain, and cognitive development (Ahmed et al., 2012; Godfrey et al., 2017; Chen et al., 2018). In this review, we address this worldwide issue associated with maternal diet by focusing on its potential long-term impact on neurodevelopment in the progeny. We strive to provide an up-to-date view of adequate nutrition during pregnancy and its effect on mothers and their offspring. Furthermore, we provide insights into diet-induced inflammatory status, microbiome as well as genetic/epigenetic changes, and their association to neurodevelopmental disorders as potential underlying mechanisms.

## Maternal Diet in Pregnancy

Even prior to conception, diet plays an important role in enabling implantation of the embryo and placentation of the future mother. Women planning for pregnancy require an increased intake of folate equal to 400 μg/day, often ingested as dietary supplement (Parisi et al., 2014), prior to conception until the 12th week of pregnancy (Plecas et al., 2014). During pregnancy, several suggested essential nutritional requirements (i.e., carbohydrates, fats, proteins, vitamins—A, B, and C—and minerals—iodine, iron, magnesium, selenium, zinc) almost double (see Table 1) (Katamay et al., 2007; Ares Segura et al., 2016; Kominiarek and Rajan, 2016; Mousa et al., 2019). This increased need mainly occurs in the second and third trimesters of gestation, i.e., the main period of fetal growth (Nnam, 2015). Then, after pregnancy, the nutrient requirements for breastfeeding mothers also differ from those in non-pregnant state; increasing for some nutrients (i.e., vitamins—A, B2, B5, B6, B8, B12, C, and E—and minerals—selenium and zinc) while decreasing for others (i.e., proteins, vitamins—B3 and B9—and minerals—iodine, iron, and magnesium) (Katamay et al., 2007; Ares Segura et al., 2016; Kominiarek and Rajan, 2016; Mousa et al., 2019).

**TABLE 1:** Selected macro- and micronutrients recommended daily consumption for non-pregnant, pregnant, and breastfeeding women (Katamay et al., 2007; Ares Segura et al., 2016; Kominiarek and Rajan, 2016; Mousa et al., 2019).

Nutrients	Recommended intake		
	Non-pregnant	Pregnant	Breastfeeding
Macronutrients			
Carbohydrates (g/day)	130	**175**	N/A
Fats and fatty acids			
*n*-6 α-Linolenic acid (g/day)	12	**13**	N/A
*n*-3 Linoleic acid (g/day)	1.1	**1.4**	N/A
Proteins (g/day)	46–50	**60–71**	**62–65**
**Micronutrients**			
**Vitamins**			
Water-soluble			
B1 (thiamin) (mg/day)	1.1	**1.4**	**1.4**
B2 (riboflavin) (mg/day)	1.1	**1.4**	**1.6**
B3 (niacin) (mg/day)	14	**18**	**17**
B5 (pantothenate) (mg/day)	5	**6**	**7**
B6 (mg/day)	1.3	**1.9**	**2.0**
B8 (biotin) (μg/day)	30	30	**35**
B9 (folate) (μg/day)	400	**600**	**500**
B12 (cobalamin) (μg/day)	2.4	**2.6**	**2.8**
C (mg/day)	75	**85**	**120**
Fat-soluble			
A (μg/day)	700	**770**	**1300**
D (μg/day)	5	5–15	5–15
E (mg/day)	15	15	**19**
K (μg/day)	90	90	90
**Minerals**			
Calcium (mg/day)	1,000	1,000	1,000
Iodine (μg/day)	150	**220–250**	**190**
Iron (mg/day)	18	**27–60**	9–27
Magnesium (mg/day)	310-320	**350–360**	310–320
Phosphorous (mg/day)	700	700	700
Selenium (μg/day)	55	**60**	**70**
Sodium (mg/day)	<2,000	<2,000	<2,000
Zinc (mg/day)	8	**11**	**12**

*N/A, not available. Increased recommended intake are highlighted in bold.*

Increased intake of macronutrients (fats, carbohydrates, and proteins) and micronutrients (vitamins and minerals) is generally recommended in the nutritional guidelines for pregnant women. In addition, guidelines exist about excluding certain food sources that may contain teratogens (substances that are known to have damaging effects on the embryo), unsafe bacteria (e.g., certain dairy or fish products), as well as avoiding alcohol and caffeine (200 mg/day) consumption (Plecas et al., 2014; Martin et al., 2016) and lowering salt (or sodium chloride) intake (Katamay et al., 2007). The so-called dietary supplements are also recommended when dietary consumption alone does not fulfill nutrient requirements, such as in women following a vegetarian/vegan diet, living in cold climates or with malabsorption disorders (Kominiarek and Rajan, 2016).

As we will further detail, overall, maternal diet is critical for the progeny’s proper development and maturation. Inadequate supply of macro- and micronutrients may cause a broad range of adverse outcomes for the fetus, ranging from premature birth and neurodevelopmental defects (neural tube, cognitive, and motor) to death (Nnam, 2015; Martin et al., 2016).

### Maternal Supplements

To support the dietary intake of pregnant or nurturing women, supplements are often recommended in nutritional guidelines, especially when the necessary nutrients cannot be fully obtained from their diet (Kominiarek and Rajan, 2016). The extent to which these supplements confer beneficial effects varies with the specific nutrients. For instance, calcium supplementation studies show reduced preeclampsia and preterm delivery in higher risk group of pregnant women without improving outcomes for the newborn (Hofmeyr et al., 2018). Similarly, zinc supplements had a small positive effect on decreasing preterm births (Donangelo and King, 2012). In contrast, supplementation trials with *n*-3-long chain poly-unsaturated fatty acids (PUFAs) during pregnancy and lactation revealed improved general cognitive score of 2–5 years old children, without significant and specific improvement reported on cognition, language, or motor development (Gould et al., 2013). For vitamin B9 supplement, studies suggest a beneficial effect mainly in decreasing risk of birth defects (Maria De-Regil et al., 2010). Vitamin B12 supplementation helps normalize maternal cholesterol plasma levels, as well as lipid metabolism in the offspring (Khaire et al., 2015b). Still, additional unbiased studies with bigger sample sizes are needed to determine the real beneficial effects of supplementation for certain nutrients such as iodine (Harding et al., 2017). Supplementations of iron and vitamin C, which can assist with iron absorption, are routinely recommended during pregnancy due to a twofold increase in need and the common occurrence of anemia during pregnancy (Kominiarek and Rajan, 2016). However, in the case of vitamin C supplementation, no significant effect has been observed on pregnancy complications (i.e., intrauterine growth restriction, preeclampsia, preterm labor, stillbirth) (Rumbold et al., 2015). Cosupplementation has been proven beneficial in certain cases, notably the cosupplementation of magnesium, zinc, and vitamin D was shown to decrease inflammation and oxidative stress in women with gestational diabetes (Jamilian et al., 2019).

Although the intake of supplements seems to have beneficial effects for the most part, a majority of the studies investigating the effects of supplements have overlooked demographic and lifestyle factors (e.g., maternal age, ethnicity, comorbidities, physical activity) that may interact in producing the reported pregnancy outcomes. Therefore, the results of these studies should be considered and interpreted with caution, and future investigation should consider demographic and lifestyle factors, together with other potential factors influencing supplements absorption and outcomes (e.g., dose, food source, and method of absorption).

### Maternal Adaptation in Pregnancy

**FIGURE 1:**
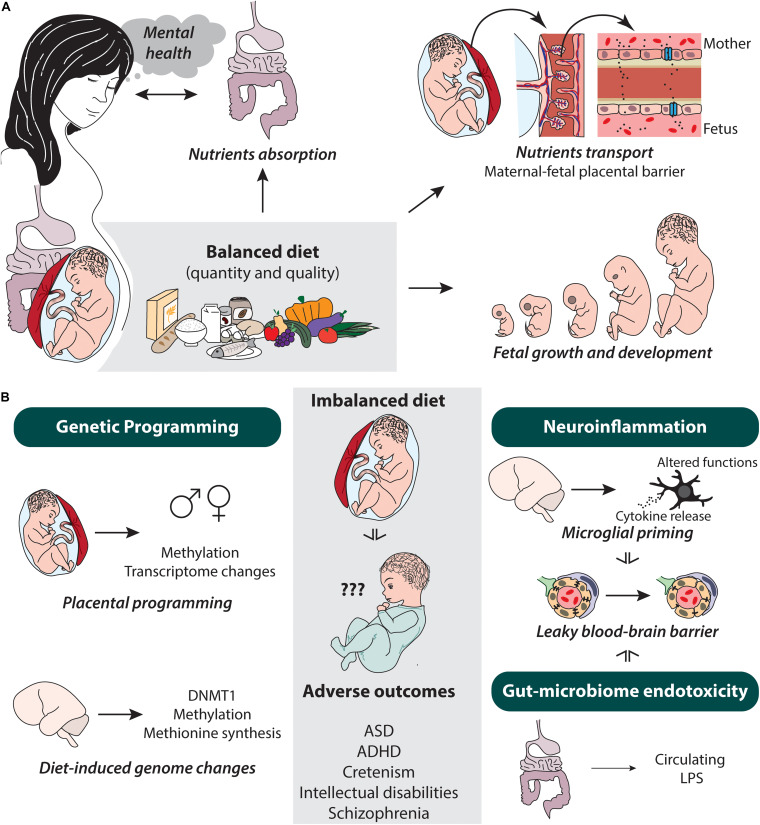
Potential dietary-mediated factors altered by the pregnant mother’s diet and putative changes and mechanism occurring in the progeny. **(A)** Balanced diet influences on nutrient absorption and transport, as well as fetal growth and development in pregnant women. Their gut microbiome can also influence mental health state of the pregnant women. **(B)** In the offspring, diet can influence genetic programming (i.e., placental and genome-wide), neuroinflammation (i.e., microglia, cytokines, and blood–brain barrier leakiness), and gut microbiome endotoxicity, which in turn causes adverse neurodevelopmental outcomes such as ASD, ADHD, cretinism, intellectual disabilities, and schizophrenia. ♂, male; ♀, female; ASD, autism spectrum disorder; ADHD, attention-deficit-hyperactivity disorder; DNMT1, DNA (cytosine-5)-methyltransferase 1; LPS, lipopolysaccharide.

Several strategies are in place within the future mother’s organism to ensure an optimal nutrition and development of the progeny (see Figure 1A). As metabolism changes during pregnancy from an anabolic (building up) to a largely catabolic (breaking down) state (King, 2000), nutrient absorption by the mother’s intestine is increased, while their excretion from the mother’s kidneys and gastrointestinal tract is altered (Zhang et al., 2013; Plecas et al., 2014). In addition to these metabolic changes, nutrients are redirected to the placenta and mammary glands, as well as mostly transferred to the developing fetus (Nnam, 2015). To accommodate this increased requirement of blood flow for nutrient and oxygen delivery to the placenta (Plecas et al., 2014; Nnam, 2015), the mother’s blood volume also increases by 35–40%, representing an expansion of 45–50% of the plasma volume and 15–20% of the erythrocyte population (Nnam, 2015).

The placenta itself is critical to nutrient transfer from the mother to the fetus, where most nutrients cross by diffusion, while other nutrients require facilitated diffusion or active transport through the placenta (McArdle et al., 2008; Desforges and Sibley, 2009; Plecas et al., 2014; Lewis et al., 2018). Nutrients, then, enable cellular growth, migration, differentiation, and fetal development, where amino acids, the building blocks of proteins, are particularly of utmost importance (Desforges and Sibley, 2009; Plecas et al., 2014) and where glucose provides about 75% of the fetus energy needs (Plecas et al., 2014).

### Absorption and the Gut Microbiome

Another important adaptation that takes place in pregnancy pertains to the microorganisms that live in our intestines and help process our dietary intake (see Figure 1A). The gut microbiome is critical for both the harvest and storage of energy sources (reviewed in Krajmalnik-Brown et al., 2012). While energy demands mainly increase during the latter part of pregnancy, adjustments begin to already take place early in pregnancy (King, 2000).

Although pregnancy is a physiological state, many of the body’s metabolic and immune adaptations resemble those of dysfunctional states in non-pregnant individuals with metabolic syndrome, such as increased energy harvest, adiposity, and decreased insulin and leptin sensitivity (Koren et al., 2012; Gohir et al., 2015b). These changes may prove necessary to provide for the high-energy demands of fetal growth during the third trimester and milk production during breastfeeding after birth. Moreover, there is some evidence that contributing to these adaptations are concomitant changes in the gut microbiome (Collado et al., 2008; Gohir et al., 2015b). Pregnancy-associated changes include a general increase in the amount of bacteria living in the gut, although phyla diversity is reduced (Collado et al., 2008). In addition, *Firmicutes* and *Bacteroidetes*, the two normally predominant phyla (90%) of the gut microbiome (reviewed in Ley et al., 2006; Rinninella et al., 2019) do not appear to undergo important alterations with pregnancy (Collado et al., 2008). However, the *Proteobacteria* phylum and *Actinobacteria* phylum (mainly *Bifidobacterium* spp.), which are respectively associated to increased inflammatory status, and immune stimulation and metabolic function, are increased from the first to the third trimesters (Collado et al., 2008; Rinninella et al., 2019).

Changes in the gut microbiome are important to consider because to meet the body’s needs, 10–30% of energy intake is harvested in the large intestine where undigested carbohydrates and proteins are further processed (fermented). With the help of the resident microbes, this fermentation process results in the production of monosaccharides and short-chain fatty acids (SCFA): primarily acetate, propionate, and butyrate in a 3:1:1 proportion (Macfarlane and Macfarlane, 2003). These metabolites have somewhat ambiguous functions, contributing to lipogenesis (monosaccharides) that can ultimately lead to the increase in adipose tissue and insulin resistance. This is in part due to an increase of circulating free-fatty acids and the proinflammatory cytokine production associated to some types of adipose tissue expansion and SCFAs propionate and butyrate (Krajmalnik-Brown et al., 2012; Gohir et al., 2015a; Jiang et al., 2019).

However, SCFAs appear to also be an important source of fuel, because of the ease with which they are absorbed through the gut by non-ionic diffusion (Kamp and Hamilton, 2006), dependent on the pH, with slight acidity, presumed from bacterial metabolic activity (Schönfeld and Wojtczak, 2016). SCFAs then travel *via* the bloodstream to the liver where they are metabolized also with relative ease not requiring protein binding, transportation, and transmembrane translocation (reviewed in detail in Schönfeld and Wojtczak, 2016). Importantly, SCFAs are not stored as adipose tissue and appear to stimulate energy expenditure as they modulate the liver’s metabolism of carbohydrates and lipids by inhibiting glucose production, glycolysis, thus contributing to prevent hyperglycemia and promoting fat oxidation (Wu et al., 2005; Gao et al., 2009). In addition, SCFAs also regulate gut satiety hormones glucagon-like peptide-1 (GLP-1) and peptide YY (PYY) and because SCFAs can cross the blood–brain barrier, they can further have direct effects in the brain and they are known to centrally stimulate another satiety hormone: leptin (Canfora et al., 2015). Lastly, SCFAs appear to have beneficial immune regulatory properties, increasing the anti-inflammatory functions of regulatory T cells in the colon (Smith et al., 2013).

In the context of pregnancy, while more research is required, all of these features of SCFAs are thought to be favorable, with high-fiber diets leading to high quantities of acetate in production and having a protective effect against asthma in the offspring (Thorburn et al., 2015). The gut microbiome is further involved in synthesis and absorption of micronutrients like vitamins and amino acids (Rodríguez-Concepción and Boronat, 2002; Bäckhed et al., 2005; Gill et al., 2006, latter reference for supplementary detailed tables). This finding proposes an additional mechanism by which altered microbiome could impact pregnancy (Gohir et al., 2015a), since there is already an increased urine excretion of water-soluble micronutrients (Ladipo, 2000) like vitamins B6, B12, folate (B9), and thiamin (B1), which are crucial for fetal development (Jans et al., 2015).

The gut microbiome increases macronutrient absorption and synthesis, which helps in building energy stores and regulates the immune system. Since pregnancy is for the most part anabolic, this is not inherently negative except when there is overconsumption of macronutrients, for instance of fats, which we will detail in section “Gut Microbiome-Mediated Endotoxicity,” and which appears to alter microbial communities in a way that impacts general metabolism and micronutrient synthesis even when fatty diets are consumed prior to conception (Gohir et al., 2015a). Notably, some metabolites that are produced by the gut microbiome can play a beneficial role in the body’s regulation of inflammation thus protecting the fetus.

### Mental Health and the Gut Microbiome

Psychosocial adaptation is another big aspect of pregnancy and that can be linked to diet and the gut microbiome. An altered gut microbiome can also affect pregnancy in another important way: through its effect on maternal mental health (see Figure 1A). It is increasingly recognized that the gut commensal bacteria have both direct and indirect effects on cognition and behavior. In a landmark paper, germ-free mice, which are born to a sterile environment and are thus not colonized by bacteria, were shown to have an inadequate development of their stress response through the hypothalamus-pituitary-adrenal axis, hence altering anxiety-like behavior (Sudo, 2014). The alterations were reversed when the mice were colonized by specific strains of “good” gut bacteria early during development but not if the intervention was done in adulthood (Sudo et al., 2004). Some animal models of depression show that microbiome is altered and in healthy human volunteers, probiotics have been shown to alleviate distress (Cryan and Dinan, 2012), and others have shown that consumption of foods that are rich in fat and/or carbohydrate alleviate anxiety (Wurtman and Wurtman, 1995; Hurley et al., 2005; Teegarden and Bale, 2008). The reality is that both anxiety and depression during pregnancy and in postpartum stages are common and can lead to adverse outcomes often encompassing preterm birth and low birth weight (reviewed in Dunkel Schetter and Tanner, 2012) as well as behavioral alterations during childhood (reviewed in Rees et al., 2019). Less understood is the degree to which general socioeconomic situation (Stringhini et al., 2010) and chronic stress contribute to the suboptimal management of pregnancy and diet (reviewed in Monk et al., 2013), which in turn, could underlie immunological and endocrine alterations, as well as anxiety and depression affecting the fetus and the maternal behaviors postpartum (Dunkel Schetter and Tanner, 2012). Notwithstanding, we know that the gut microbiome is critically involved in modulating the stress and immune response, which are important features of both anxiety and depressive disorders (reviewed in Morris et al., 2017; Peirce and Alviña, 2019).

## Specific Nutrient Imbalance and Inflammation

### Macronutrients

Inappropriate availability of macronutrients—in deficiency or excess—can have long-term effects on the development of several body systems of the progeny (e.g., metabolic, circulatory, pulmonary). Protein is a macronutrient that when insufficiently consumed can lead to severe developmental consequences, including intrauterine growth restriction, impaired brain growth, and neurocognitive deficits (Monk et al., 2013). Meeting adequate protein requirements during pregnancy is most important during the second and third trimesters, when growth and development of both maternal and fetal tissues is accelerated (Kominiarek and Rajan, 2016). Studies on rodent adult offspring have demonstrated that low protein intake during pregnancy led to macrostructural changes of the brain such as decreased cerebrovascular density (see Table 2) (Bennis-Taleb et al., 1999). However, carbohydrate and fat consumption has become a pressing nutritional focus as worldwide, millions of pregnant women in developed countries suffer from obesity or overweight (Chen et al., 2018) mainly due to excessive carbohydrate and fat intake (Bleich et al., 2008). Critically, some of its negative consequences are tied to inflammation (Bolton and Bilbo, 2014; Buyken et al., 2014; Castanon et al., 2015; Ludwig et al., 2018; Montalvo-Martínez et al., 2018).

**TABLE 2:** Maternal and fetal outcomes of essential nutrient imbalance.

Nutrients	Imbalance	Outcomes		References
		Mother	Fetus/child	
Proteins	↓	N/A	Brain structural changes ↓ cerebrovasculature	Bennis-Taleb et al., 1999
Carbohydrates	↑	↓ hypertension ↓ adverse outcomes	↓ adverse outcomes	Gabbe and Quilligan, 1977; Kattah and Garovic, 2013; Sanjarimoghaddam et al., 2019
	↓ or ↑↑↑	Metabolic disruptions	Metabolic disruptions, death, stillbirth	Koski et al., 1986; Yamashita et al., 2000
Fatty acids (*n*-6/*n*-3)	↓ *n*-3	↑ postpartum depression	N/A	Horvath et al., 2007; Kim et al., 2017; Hoge et al., 2019
	↑ *n*-3	↓ preterm delivery in high-risk pregnancy	N/A	
	↑↓ *n*-6/*n*-3	N/A	Altered neurodevelopment	
Fats (saturated and unsaturated)	↑	Obesity, overweight, diabetes, restricted intrauterine growth, preeclampsia, C-section	Stillbirth, metabolic disorders, altered behaviors (anxiety, cognitive, social, motor)	Bilbo and Tsang, 2010; Sullivan et al., 2010, 2015; Tozuka et al., 2010; Peleg-Raibstein et al., 2012; Sasaki et al., 2013; Bolton and Bilbo, 2014; Castanon et al., 2015; Grissom et al., 2015; Tarrade et al., 2015; Graf et al., 2016; Ohta et al., 2017; Montalvo-Martínez et al., 2018; Thompson et al., 2018; Winther et al., 2018; Cordner et al., 2019; Smith et al., 2019
Ketone bodies	↑	Mortality, morbidities in the long term (cardiac, gastrointestinal, renal complications)	Growth alteration, altered behaviors (anxiety, ADHD, cognition, depression)	Rizzo et al., 1991; Sussman et al., 2013, 2015; von Geijer and Ekelund, 2015; Kanikarla-Marie and Jain, 2016; Nnodum et al., 2019
Vitamin A	↓	Night blindness, anemia, ↓ immune responses	Death, delayed growth, fetal malformations	Sommer and Davidson, 2002; Spiegler et al., 2012; Bastos Maia et al., 2019
	↑	Miscarriage	Fetal malformations	
Vitamin B9 (folate)	↓	Miscarriage, restricted intrauterine growth, preeclampsia	Fetal malformations	Fekete et al., 2012
Vitamin B12 (cobalamin)	↓	Preterm delivery	Anemia, fetal malformations, and altered behaviors (cognitive, motor)	Pepper and Black, 2011; Rogne et al., 2017; Sayar et al., 2020
Vitamin D and calcium	↓	Implantation failure, placental insufficiency, diabetes, miscarriage, preterm delivery, preeclampsia, C-section, poor immune response/tolerance	Fetal malformation, metabolic disorders (diabetes, hypertension, stroke, coronary artery disease), atopic disorders (asthma, eczema, hay fever), CNS disorders (ASD, multiple sclerosis, schizophrenia)	Merewood et al., 2009; Shin et al., 2010; Heyden and Wimalawansa, 2018
Sodium chloride (salt)	↑	Hypertension, restricted intrauterine growth, placental abruption, preeclampsia, comorbidities (cardiovascular, renal, hepatic CNS disorders)	Death, growth delay, cardiovascular, renal, CNS disorders (ASD, ADHD, schizophrenia)	Kattah and Garovic, 2013; Kleinewietfeld et al., 2013; Mao et al., 2013; Ha, 2014; Choe et al., 2015; Seravalli et al., 2016; Stocher et al., 2018; Afroz and Alviña, 2019; Faraco et al., 2019; Riise et al., 2019; Lahti-Pulkkinen et al., 2020
Iodine	↓	Hypothyroidism	Death, growth delay, cretinism, hypothyroidism, goiter	Skeaff, 2011; Kominiarek and Rajan, 2016; Pearce et al., 2016
	↑	Iodine-induced hypothyroidism, hyperthyroidism	N/A	Pearce et al., 2016
Iron	↓	Anemia, placenta hypertrophy, inflammation, poor milk quality	Death, anemia, growth delay, metabolic disorders (obesity, high blood pressure), altered neurodevelopment (neurotransmitter metabolism, neurotransmission, myelination), altered behaviors (cognitive, motor, emotive, psychology)	O’Connor et al., 1988; Huang et al., 2001; Gambling et al., 2002, 2004; Lozoff and Georgieff, 2006; Alwan and Hamamy, 2015; Means, 2020
Zinc	↓	Preterm delivery, prolonged labor, hypertension, increased risk of infection	Death, fetal malformations, growth delay, seizure, altered behaviors (anxiety, hypotonia, ADHD, social deficits)	Donangelo and King, 2012; Roohani et al., 2013; Grabrucker et al., 2014, 2016; Sauer and Grabrucker, 2019

*↓, reduced; ↑, increased; ↑↓, imbalance; ↑↑↑, excessive; ASD, autism spectrum disorders; ADHD, attention-deficit-hyperactivity disorders; C-section, cesarean section; CNS, central nervous system; N/A, not available.*

#### Carbohydrates and Fats

Carbohydrates are involved in several important bodily processes, including energy supply, glycemia control, insulin metabolism, cholesterol and triglyceride metabolism (Slavin and Carlson, 2014; Holesh et al., 2020), as well as maintenance of the gut microbiome’s health and diversity (Turnbaugh and Gordon, 2009). They are mainly found in whole-grain foods (Plecas et al., 2014) and also in fruits, vegetables, and milk products (Slavin and Carlson, 2014; Holesh et al., 2020). Carbohydrates are classified into three main categories: sugars (i.e., simple sugars like mono- or disaccharides and complex sugars like oligo- and polysaccharides), starches (i.e., complex polysaccharides made of several glucose molecules produced by plants), and fibers (i.e., non-digestible complex carbohydrates) (Slavin and Carlson, 2014; Holesh et al., 2020). Carbohydrates act as the main energy source for maintaining pregnancy and lactating processes, the offspring’s growth and development (Plecas et al., 2014; Martin et al., 2016), as well as milk production (Ares Segura et al., 2016; Martin et al., 2016) (see Table 2).

The type of carbohydrate has been shown to determine different inflammatory properties. In fact, meta-analysis looking at high-sensitivity C-reactive protein (CRP) and interleukin (IL)-6, which are inflammatory signals of the immune system, in human participant from countries across the world, have revealed a significant correlation between fiber intake and an anti-inflammatory effect, whereas whole grain was associated with an increase of inflammatory markers (Buyken et al., 2014).

Dietary recommendations for global fat intake, both saturated and unsaturated, are mainly addressed to non-pregnant women. While dietary recommendations have been made for certain essential fatty acids, α-linolenic acid (*n*-6 PUFA) and linoleic acid (*n*-3 PUFA) in particular, which are needed more in pregnancy (see Table 1) (Mousa et al., 2019), special considerations need to be taken in terms to quantity, as well as type of fats consumed during pregnancy.

Diets with high levels of fats (regardless of their saturation state) can induce maternal overweight or obesity and diabetes, notably by sustaining a proinflammatory state, as demonstrated in diverse animal models of maternal high-fat diet (mHFD) (Bolton and Bilbo, 2014; Castanon et al., 2015; Montalvo-Martínez et al., 2018). Beyond the increased fat mass, rodent models have also revealed an inflammatory state associated to mHFD (e.g., increase of cytokines IL-2, IL-4, IL-6) (Castanon et al., 2015; Graf et al., 2016; Bordeleau et al., 2020; Kretschmer et al., 2020). Indeed, *in vitro* experiments confirmed that IL-6 can promote apoptosis of endothelial cells, thus impairing placental vasculature and leading to intrauterine growth restriction *in vivo* in a mHFD mouse model (Kretschmer et al., 2020). Moreover, mHFD can also lead to behavioral changes in the mothers inducing increased anxiety-like behaviors (Thompson et al., 2018), which can negatively impact offspring’s maternal care (Reck et al., 2018) (further detailed in Table 2). Prospective longitudinal study on pregnant women revealed that under stressful-perceived situations, women tend to consume more proinflammatory diet, such as a diet high in fats (Lindsay et al., 2018). Considering that more than three quarters of pregnant women experience low to moderate mood during gestation (Woods et al., 2010), high fat consumption during pregnancy may be more prevalent than we conceive.

One essential type of fat, especially required during pregnancy and breastfeeding, is the so-called PUFAs. In the maternal diet, omega 3 (*n*-3) PUFAs are found in fish oil or linseed oil, where fish source of *n*-3 PUFA is notably more efficient at providing necessary PUFAs to the progeny’s brain (Fernandes et al., 2012). PUFAs are formed by an acyl chain of at least 18 carbons with one acid (–COOH) end and one methyl (–CH_3_) end (Fernandes et al., 2012). Essential PUFAs include *n*-3 PUFAs linoleic acid and omega 6 (*n*-6) PUFAs α-linolenic acid. These two PUFAs can synthetize other PUFAs of the same unsaturation class (*n*-3 or *n*-6, indicating the position of the first double bound, C = C, on the chain). Since *n*-3 and *n*-6 compete for the enzymes desaturases and elongases, dietary intake of linoleic acid and α-linolenic acid influences their conversion rate (Taha et al., 2014; Bentsen, 2017). During the third trimester of pregnancy up to the first 2 years after birth in humans, arachidonic acid (*n*-6 PUFAs) and docosahexaenoic acid (*n*-3 PUFAs) are the most abundant PUFAs measured in the brain (Hadley et al., 2016). During this time, PUFAs have been shown to contribute to brain growth (Hadley et al., 2016), neurogenesis (Kawakita et al., 2006; Tokuda et al., 2014), synaptic plasticity, and neuronal wiring in animal and clinical human studies (Owens and Innis, 1999; Hamazaki et al., 2005; Darios and Davletov, 2006). Of note, *n*-6 PUFA is less reliant on dietary intake than *n*-3 PUFA (Taha et al., 2014).

*n*-3 PUFAs, like docosahexaenoic acid, can directly interact with transcription factors involved in inflammatory processes—nuclear factor-κB (NFκB) or peroxisome proliferator-activated receptor γ (PPARγ)—and thus, modulate the maternal and placental inflammatory status. Although the specific underlying mechanism remains under investigation, it was postulated that *n*-3 PUFAs might act on lipid mediators and help maintain placental functions during pregnancy through their anti-inflammatory properties (Akerele and Cheema, 2016; Lewis et al., 2018). Supplemental intake of docosahexaenoic acid in male mouse fed with a diet high in fats was also shown to soothe hypothalamic high-fat diet (HFD)-induced inflammation by decreasing suppressor of cytokine signaling 3 (SOCS3) signaling and promoting the Janus kinase (JAK)/protein kinase B (Akt) pathway. Other than its action on inflammation, *n*-3 PUFAs taken in this context helped to normalize the metabolic energy-balance (Cheng et al., 2020). However, imbalance of PUFAs such as excess of *n*-3 PUFAs, may inhibit the production of crucial proinflammatory cytokines during gestation (Akerele and Cheema, 2016). Cyclooxygenases and lipoxygenases can convert PUFAs into short-lived hormones—eicosanoids—that possess inflammatory properties (e.g., prostaglandins, thromboxanes, lipoxins, and leukotrienes). In animal model and human studies, *n*-6 PUFAs-derived eicosanoids have been commonly described as proinflammatory, however, they can also contribute to inflammatory resolution, while *n*-3-derived eicosanoids are anti-inflammatory (Phillis et al., 2006; Calder, 2009; Innes and Calder, 2018). High dietary intake of *n*-6 PUFAs has been long believed to be linked to heightened inflammation but, enhanced inflammation was not consistently observed in different human studies (Innes and Calder, 2018). Excessive consumption of fats and/or sugar, a hypercaloric diet, can also promote a proinflammatory status in the pregnant woman or nurturing mother (Montalvo-Martínez et al., 2018).

Another important contribution of dietary fats during pregnancy is in the production of ketone bodies. Fatty acids can be broken down in the liver into ketone bodies—3-hydroxybutyrate and acetoacetate—(Paoli et al., 2013; Paoli, 2014), which are distributed throughout the body as a metabolic substrate, i.e., as fuel instead of glucose. With their metabolic changes during gestation and lactation, including reduced insulin sensitivity, women usually have an elevated level of circulating ketones (Paterson et al., 1967; Nnodum et al., 2019). Ketone bodies pass freely through the placenta or mother’s milk, and they provide supplemental energy to the developing fetus. For the developing central nervous system (CNS), ketones not only can act as an energy source but also be used in lipogenesis as a lipid precursor. Moreover, ketones can modulate CNS functions, notably by partaking in adenosine triphosphate (ATP) synthesis and carbon pathway (Edmond et al., 1985; Williamson, 1985; Bronisz et al., 2018). While ketones possess important roles for fetal and neonate development, the consumption of a ketogenic diet and its implication during pregnancy is complex and it remains largely unclear whether it is beneficial or detrimental.

#### Ketogenic High-Fat Diet

One HFD that gained a lot of popularity in the past years is the ketogenic diet, initially used for its anticonvulsant and protective effects in neurodevelopmental disorders (Yudkoff et al., 2007). With ketone-induced metabolic changes, ketogenic diet have been suggested to alleviate symptoms or features when consumed by individuals with neurologic or neuropsychiatric disorders like autism spectrum disorder (ASD), epilepsy, or schizophrenia (Yudkoff et al., 2007; Kraft and Westman, 2009; Gano et al., 2014; Ruskin et al., 2017a,b). In addition, it is also known for its relatively rapid effects in reducing weight (e.g., obesity), inflammation, and metabolic alterations (Ludwig, 2020). Over the years, variations of ketogenic diets have been proposed (Shilpa and Mohan, 2018) but typically it involves 80–90% of the calories coming from lipids (high-fat/low-carbohydrate, moderate proteins). Upon 3–4 days of fasting or ketogenic diet intake, there is an increased production of ketone bodies and metabolism shifts from glycolysis to ketosis in several organs including the brain (Yudkoff et al., 2007). Mild ketosis is a physiological process that is known to be induced in fasting, lactation, shortly after exercising or muscular activity (Dashti et al., 2004). In fact, mild ketosis was a “normal” metabolic-state preagriculture, and it is still observed in some populations (e.g., Inuit in the Artic, First Nation groups in Canada) (Ludwig et al., 2018). Of note, ketosis is different from ketoacidosis, which can occur in pathological conditions such as uncontrolled type 1 diabetes, where a lack of insulin prevents most organs from using the available glucose, this leads to ketone bodies being produced in excess of 20 mmol/L while the body attempts to eliminate excess glucose *via* urine, causing a lowering of the pH of the blood and dehydration with potentially fatal consequences (Mullins et al., 2011; Paoli, 2014).

While ketogenic diet improves insulin sensitivity in non-pregnant individuals (Dashti et al., 2004), it was suggested that ketogenic diet might not be as beneficial for pregnant women and their progeny (see Table 2) (von Geijer and Ekelund, 2015; Kanikarla-Marie and Jain, 2016; Nnodum et al., 2019). Indeed, the outcome of a ketogenic diet on the offspring is complex; it may also differ according to the type of fats consumed, which change the gut microbiome: high proportions of saturated and monounsaturated fats appear to have a negative impact on its diversity while high polyunsaturated does not (Paoli et al., 2019; Wolters et al., 2019). This can then impact metabolic and inflammatory status and underlying health conditions (e.g., overweight, diabetes, neurological, neuropsychiatric) of both the mother and the developing fetus.

In contrast to a conventional HFD, ketogenic HFD has been proposed to induce more of an anti-inflammatory profile in non-pregnant individuals. In fact, ketogenic diets have reported a decrease in cellular stress by reducing reactive oxygen species production and enhancing antioxidant activities, as well as elevating circulating levels of PUFAs through the increased activity of fatty acids oxidation (Gano et al., 2014). In the context of maternal immune activation (MIA), a postnatal ketogenic diet in the offspring demonstrates a protective effect (Ruskin et al., 2017b); however, it remains to be investigated if this protective effect on the postnatal brain is due to anti-inflammatory and metabolic modulation by the ketones and/or acting *via* the gut microbiome (Ang et al., 2020).

### Micronutrients

Other important considerations during pregnancy pertain to deficiency in micronutrients such as vitamin A (Garcia−Casal et al., 2018), vitamin B9, vitamin B12, vitamin D, calcium, iodine, iron, or zinc, among others (Blumfield et al., 2013; Visentin et al., 2016; Garcia−Casal et al., 2018). Maternal imbalance or inappropriate intake can lead to detrimental outcomes for both the mother’s pregnancy (e.g., preeclampsia, intrauterine growth restriction) and the offspring’s development (e.g., stillbirth, growth delay, risk of developing disorders detailed in Table 2 for each nutrient). Among those essential micronutrients, several share inflammatory properties, which, in the context of pregnancy, could contribute to the detrimental outcomes on both pregnancy and progeny.

#### Vitamin A

Vitamin A is an essential nutrient found in food from animal sources like dairy products, liver, and fish oil, as well as in food from vegetal sources (e.g., fruits, leaves, tubers). Vitamin A from vegetal sources is poorly absorbed, however, compared with animal sources (Bastos Maia et al., 2019). Vitamin A is involved in several physiological functions through its active oxidized forms: retinaldehyde and retinoic acid. Retinaldehyde is involved in visual function, whereas retinoic acid can act as ligand for the nuclear retinoic acid receptor and regulate the transcription of genes involved in reproduction, development, growth, and immunity. During pregnancy, vitamin A and its derived products are needed by the mother for placental maintenance and by the embryo for the formation and development of various organs (i.e., hearth, eye, kidney, lung, limbs, spinal cord, and brain). The placenta stores vitamin A that mobilizes to the fetus during prenatal development. This storing process ensures an adequate delivery of retinoids in cases of maternal insufficient intake to protect the developing fetus (reviewed in Spiegler et al., 2012).

Vitamin A through its metabolized active form, retinoic acid, can modulate immune homeostasis by binding to retinoic acid and retinoid receptors, which then acts as and interacts with transcription factors (Spiegler et al., 2012; Oliveira et al., 2018). As such, retinoic acid can modulate inflammatory processes—including infiltration of immune cells, production of cytokines [e.g., IL-1β, IL-4, IL-6, IL-10, IL-12, IL-18, interferon (IFN)-γ, transforming growth factor beta (TGF-β), tumor necrosis factor (TNF)-α], and other inflammatory mediators [NFκB, NOD-like receptor family pyrin domain-containing protein 3 (NLRP3)]—in a variety of tissues (Kang et al., 2000; Wang et al., 2007; Oliveira et al., 2018; Elshal et al., 2019; Alatshan et al., 2020). Thus, dietary intake of vitamin A can influence inflammatory response. For instance, in the context of dermatitis, low vitamin A exacerbates its severity partially through an increase of T cell release of immunoglobulins (i.e., IgG1, IgE) and cytokines (i.e., IL-4, IL-13) (Yang et al., 2020).

Other than immune mediators, vitamin A can modulate the integrity of the intestinal barrier by promoting expression of tight junction proteins (i.e., claudin-1, occludin, zonula occludens-1) (He et al., 2020). By doing so, it may modulate trafficking of metabolites coming from the diet or produced by the gut microbiome. In pregnancy, this could imply that vitamin A can influence maternal and fetal outcomes directly on the immune system or indirectly through the gut-immune axis.

Surprisingly, in a recent study on ulcerative colitis, it was demonstrated that increased levels of retinoic acid are associated with higher levels of proinflammatory cytokines (i.e., IL-17, INF-γ) and lower levels of anti-inflammatory cytokines (i.e., IL-10) in the intestinal mucosa of patients. It was postulated by the authors that in the presence of inflammation, retinoic acid maintains inflammation by upregulating proinflammatory molecules (Rampal et al., 2020). Therefore, it seems that the role of vitamin A during inflammatory processes is complex and may be modulated by the diet and interacts with the inflammatory state of the person, whether it is a chronic inflammatory disorder or an immune-privileged state like pregnancy.

#### Essential Vitamin B

Vitamin B9 or folate is a water-soluble B vitamin found in green-colored vegetables and citrus fruits. Its synthetic form—folic acid—is most stable when used in supplements (Kominiarek and Rajan, 2016). Folate itself is involved in the synthesis of DNA, RNA, and some amino acids (Fekete et al., 2012; Stamm and Houghton, 2013; Kominiarek and Rajan, 2016) as well as methylation reactions (Stamm and Houghton, 2013). Therefore, folate is important during periods of placentation, implantation of the embryo, embryogenesis, and fetal growth (Maria De-Regil et al., 2010; Parisi et al., 2014; Kominiarek and Rajan, 2016). During embryogenesis and fetal growth, the need for folate is highly increased reaching 600 mg/day (from 400 mg/day in non-pregnant women) (Kominiarek and Rajan, 2016).

Vitamin B12 or cobalamin acts as a cofactor with folate in DNA methylation (Pepper and Black, 2011; Khaire et al., 2015a,b). Cobalamin is also involved in lipid metabolism (Khaire et al., 2015a,b). B12 deficiency during pregnancy can arise in cases of women with vegetarian or vegan diets, as well as women with intestinal diseases that result in a malabsorption condition (Rogne et al., 2017).

Vitamin B insufficiency has been associated with higher levels of neuroinflammation and oxidative stress, while supplementation of vitamin B reduces oxidative stress and inflammation by increasing oxidative metabolism that may promote energy storage and developmental processes (Ford et al., 2018).

#### Vitamin D and Calcium

Vitamin D and calcium are closely related in terms of their metabolism. Obtained either through dietary consumption or mostly synthetized by the skin in contact with sunlight, the active form of vitamin D promotes calcium absorption (Curtis et al., 2014; Kominiarek and Rajan, 2016). Dietary sources of vitamin D include eggs and fish or commonly supplemented juices and milks (Kominiarek and Rajan, 2016). Proper absorption of both vitamin D and calcium are critical to bone growth and calcification (Curtis et al., 2014; Kominiarek and Rajan, 2016), immune and inflammatory functions, as well as cellular differentiation (Kominiarek and Rajan, 2016). In the embryo, the vitamin D and calcium needs increase during the main periods of skeleton formation and calcification, which start at the beginning of the embryonic stage (formation of a cartilaginous skeleton) and end during the last trimester of pregnancy (ossification of the skeleton) (Curtis et al., 2014). Pregnant women with vegan or vegetarian dietary habits as well as woman living in cold climate (Kominiarek and Rajan, 2016; Zhou et al., 2017) or with darker skin have a higher risk of vitamin D and calcium deficiency (Kominiarek and Rajan, 2016).

Vitamin D possesses a key role in the suppression of inflammation. Indeed, *ex vivo* placental experiments demonstrated that treatment with different forms of vitamin D, 25OHD3, or 1,25(OH)_2_D_3_, attenuates lipopolysaccharide (LPS)-induced inflammation (Liu et al., 2011). Vitamin D can also modulate proliferation, differentiation, survival, maturation, and cytokine release of several immune cells including dendritic cells, macrophages, T cells, and B cells (Guillot et al., 2010).

On the contrary, depletion of vitamin D receptor or hydroxylase Cyp27b1 exacerbates inflammatory mediator levels (Liu et al., 2011). Low intake of vitamin D additionally promotes a proinflammatory status, due to the reduced vitamin D-induced inhibitory action on the adaptive immune response and inflammation (Shin et al., 2010).

#### Salt

Salt or sodium chloride is easily obtained in western diet with processed food often enriched in salt (Ha, 2014). However, excessive consumption of sodium chloride can cause renal (Ha, 2014), cardiac diseases, including hypertension (Ha, 2014; Choe et al., 2015), CNS disorders (Kleinewietfeld et al., 2013; Faraco et al., 2019), and inflammation (Kleinewietfeld et al., 2013). *In vitro* studies helped to clarify the inflammatory properties of salt. For instance, sodium chloride-hypertonic stress can act as a chemoattractant to immune cells like macrophages, thus modulating their migration and mobility (Müller et al., 2013). Human and mouse macrophages treated *ex vivo* with high concentration of salt possessed a proinflammatory signature, both at the gene and protein levels, which was exacerbated following immune challenges induced by LPS (Zhang et al., 2015).

In *in vivo* studies in rodent models and humans, elevated consumption of salt was shown to promote immune activities of macrophages (Zhang et al., 2015; Guo et al., 2018), T cells (Guo et al., 2018) and dendritic cells (Xiao et al., 2020), which in turn exacerbated the onset of immune diseases (e.g., colitis, *lupus erythematosus*, lung injury) (Zhang et al., 2015; Guo et al., 2018; Xiao et al., 2020).

In maternal adipose tissue, high-salt diet increases the expression level of inflammatory molecules [e.g., IL-1β, TNF-α, *cluster differentiation* (*Cd*) *68*] in mice (Reynolds et al., 2014). In another independent study in mice, elevation of inflammatory gene expression was also reported in macrophages from the lungs [i.e., *C-X-C chemokine ligand 1* (*Cxcl1*), *Il6*, inducible nitric oxide synthase (*iNOS*)] and kidneys (i.e., *iNOS*), but not from the brain or adipose tissue (Zhang et al., 2015). Controversially, immunosuppression properties of high-salt diet—such as inhibition of IFN-γ/JAK/signal transducer and activator of transcription (STAT) pathway—was recently reported in mouse kidney cells (Arai et al., 2017). It thus seems that salt inflammatory properties may vary depending on the cell type studied. Current knowledge into the maternal-fetal effect of high-salt diet during pregnancy and lactation limits our capacity to assess the extent of salt-induced inflammatory changes in the context of maternal diet as well as neurodevelopmental disorders.

#### Minerals: Iodine, Iron, and Zinc

Together with iron and zinc, iodine is one of the minerals most commonly found deficient in the diet of pregnant women (Garcia−Casal et al., 2018). Iodine is a critical element of thyroid hormone synthesis found in seafood products as well as fortified iodized salt (Kominiarek and Rajan, 2016; Pearce et al., 2016). Thyroid hormones are important for fetal and newborn neurodevelopment by modulating cellular migration and differentiation, synaptogenesis, as well as myelination (Bernal, 2007). Maternal iodine consumption is thus critical for the fetus until its own thyroid begins producing thyroid hormones, around the second trimester, and even at this stage the fetal storage is limited until birth (Skeaff, 2011). Iodine can also act as an antioxidant (Aceves et al., 2013). On the other hand, excessive intake of iodine increases the risk of developing autoimmune thyroid disease (Luo et al., 2014), meaning that the inflammatory properties of iodine may be more complex and need further study.

Iron is important for blood cell’s ability to transport oxygen around the body. It can be found in food as two distinct forms: heme—hemoglobin and myoglobin found in meat and fish—and non-heme—obtained from cereals, fruits, and vegetables (Abbaspour et al., 2014). Nutrients can modulate its absorption; while vitamin C promotes iron absorption, milk and tea inhibit its absorption (Kominiarek and Rajan, 2016). With the increase in blood volume as well as iron-dependent developmental mechanisms during pregnancy (Nnam, 2015), iron intake is key (Means, 2020). Failing to meet the iron needs can cause, in the pregnant woman, inflammation (Gambling et al., 2002) among other detrimental outcomes on pregnancy (O’Connor et al., 1988; Huang et al., 2001; Means, 2020).

Iron intake can modulate inflammatory processes, and when intake of iron is insufficient, animal models (e.g., rodent, fish) have demonstrated that it causes a reduction of anti-inflammatory cytokines (e.g., IL-4, IL-10, IL-11, IL-15, TGF-β) and mediators [e.g., inhibitor of NFκB (IκB) α], as well as upregulation of proinflammatory cytokines (e.g., IL-1β, IL-8, IL-12, IL-17, IFN-γ) and other mediators [e.g., NFκB, IκB kinase (IKK) α/β, eukaryotic translation initiation factor 4E-binding protein 1 (4E-BP)] in the periphery (e.g., gut, placenta) (Gambling et al., 2002; Guo et al., 2019). Therefore, iron intake can modulate inflammatory states of pregnant or breastfeeding women. Iron deficiency is the most common nutrient deficiency (Stoltzfus, 2003). Further assessment particularly during the early life period, including pregnancy and childhood (McCann et al., 2020; Means, 2020) is thus warranted, considering that the severity of iron deficiency may be time sensitive (Gambling et al., 2004).

Zinc is found in red meat, seafood, and grains (Saper and Rash, 2009). Proteins generally promote zinc absorption and bioavailability (Roohani et al., 2013). Zinc-dependent enzymes, factors, or transporters are necessary for a broad range of cellular processes during division, differentiation, and function (Donangelo and King, 2012). Zinc is thus crucial to embryogenesis, fetal growth, and development, as well as milk production (Donangelo and King, 2012; Roohani et al., 2013). Zinc is also an essential mineral for intestinal microbiome flora health. In a mouse model and clinical human studies, zinc deficiency during pregnancy was shown to alter the composition of the intestinal microbiome and gut permeability (Sauer and Grabrucker, 2019), as well as promote systemic inflammation (Wang et al., 2015) and neuroinflammation (Sauer and Grabrucker, 2019). It was suggested that alteration of the gut-brain axis may directly contribute to increasing inflammatory signaling upon zinc deficiency (Sauer and Grabrucker, 2019).

Zinc can act as an inflammatory regulator. Indeed, zinc ions can inhibit signal transduction (e.g., NFκB, IFN-λ3), which in turn prevents cytokine production [e.g., IL-1β, IL-6, monocyte chemoattractant protein 1 (MCP-1), TNF-α] (Jarosz et al., 2017; Read et al., 2017; Olechnowicz et al., 2018). Zinc closely regulates zinc-dependent proteins, including A20 zinc finger protein, metalloproteinase (MMP)2, MMP9, and PPAR-α, that can contribute to inflammatory processes (Jarosz et al., 2017; Olechnowicz et al., 2018). Zinc is also critical for membrane barrier maintenance and function, where a lack of zinc can damage the membrane barrier (e.g., epidermal, gastrointestinal, pulmonary) hence permitting the entry of pathogens or toxins into the bloodstream. Moreover, zinc promotes cellular adhesion and migration (Jarosz et al., 2017). It is a key nutrient to inflammatory processes and contributes to the maintenance of immune cell homeostasis in steady-state and during pathogen-induced immune challenge (Gao et al., 2018). During pregnancy, low zinc levels increase inflammation as demonstrated by the enhanced expression of IL-6 and astrogliosis in the brain of pregnant mice fed with a zinc-deficient diet for 5 weeks prior to pregnancy and throughout gestation (Sauer and Grabrucker, 2019). This inflammation might also be involved in the development of autistic-like behavior in the offspring (Sauer and Grabrucker, 2019), including increased anxiety, impaired social behaviors (Grabrucker et al., 2014, 2016), attention-deficit-hyperactivity disorder (ADHD), hypotonia, and increased risk of seizure in later life (Grabrucker et al., 2014).

## Implication for Pregnancy and the Progeny

Together, several nutrients can directly or indirectly influence the immune system, thus potentially disturbing pregnancy and fetal development when taken in inadequate quantity or balance. Moreover, pregnancy represents a unique immunological paradox; the maternal immune system tolerates the fetus and circulating fetal antigens, while fetal trophoblast (from the outer layer of the blastocyst or embryo) invades into the maternal uterus to coordinate nutrient delivery. To allow for proper immune tolerance, several mechanisms occur within the placenta: immunosuppression by paracrine signaling, circulation of fetal cells into the maternal circulatory system, secretion of immunosuppressing molecules by trophoblast and low antigen presentation by trophoblasts. Disturbance of the immune tolerance process provokes obstetric complications for the mother and developmental alterations for the progeny (Hsiao and Patterson, 2012).

Furthermore, MIA is a well-known and characterized risk factor of neurodevelopmental disorders like ASD and schizophrenia (Mattei et al., 2014; Fernández de Cossío et al., 2017b; Beversdorf et al., 2018; Bilbo et al., 2018; Prins et al., 2018; Bordeleau et al., 2019). This inflammatory-mediated mechanism is likely behind part of the detrimental effects of an inadequate maternal diet on the offspring.

### Maternal Diet and Its Link to Neurodevelopmental Disorders

Genome-wide association studies and linkage studies have shown that the genetic architecture of many neurodevelopmental disorders comprises hundreds of genes affected, but each contributing only small effects to the overall phenotype or alternatively a single genetic mutation with large effects leading to very rare genetic syndromes (Sullivan et al., 2012). These genetic studies have often found common genetic vulnerability across diagnostically different neurodevelopment and neuropsychiatric disorders with convergent disruption of biochemical pathways that sustain synaptic and immune homeostasis (Garbett et al., 2008; Neale et al., 2012; Banerjee et al., 2014; Estes and McAllister, 2015). These pathways can, however, also be disrupted by a number of environmental factors that have now been linked to neurodevelopmental disorders. Some factors include gestational diabetes, maternal age and obesity, autoimmunity, and infection, with the common denominator between these factors being inflammatory processes during pregnancy (Estes and McAllister, 2015). The mother’ immune activation during pregnancy appears to be an important risk factor for the neurodevelopmental alterations observed in the progeny that later manifest as behavioral disorders (Boksa, 2010; Knuesel et al., 2014; Ziats et al., 2015).

Pregnancy requires a tight regulation of the maternal immune system: it must allow the implantation and growth of a partially foreign body (the fetus), while simultaneously protecting it against pathogens to ensure the conservation of species (Mor and Cardenas, 2010; Racicot et al., 2014). Diet, even before conception, can affect pregnancy and alter the inflammatory status of the mother during pregnancy, conferring greater risk to the fetus beyond intrauterine developmental stage and throughout the progeny’s developmental process. Deficiencies in micronutrients have previously been reported to have adverse outcomes for neurodevelopment. Among them, folate and vitamin B12 are important for DNA methylation, and their deficits were associated to neural tube defects (Molloy et al., 2008). In a meta-analysis examining how nutrition impacts on ASD, ADHD, and intellectual disability, folic acid and vitamin supplementation in the mother during gestation was inversely associated to neurodevelopmental disorders, particularly when supplementation occurred during early pregnancy (Li et al., 2019). Zinc is also important for neuronal development (Anjos et al., 2013) and iodine deficiency has long been known to cause cretinism, a developmental condition that has associated intellectual disability and is preventable (Cao et al., 1994). Notably, deficiency in dietary factors have also been linked to schizophrenia, as shown in three epidemiological studies focusing on specific periods of famine and reviewed in detail in association to other studies on deficient vitamin D, folate, and iron that is associated with an increased risk for this disorder (McGrath et al., 2011). Together, maternal diet has risen as an important risk factor for neurodevelopmental disorders, such as ASD, ADHD, and schizophrenia.

Protein is a macronutrient that has long been associated with impairing brain growth and thus having broad neurocognitive effects (Monk et al., 2013). Another macronutrient: fat, is also known to have important effects on neurodevelopment as components of omega-6 and omega-3 fatty acids (PUFAs) are necessary parts of neural cell membranes, and supplementation during pregnancy in controlled human trials showed better neurocognitive performance in the children (Helland et al., 2003), although supplementation with PUFAs was inconclusive in a meta-analysis looking at nutrition impacts on ASD, ADHD, and intellectual disability (Li et al., 2019). Equally important is the evidence in mice showing that diets high in saturated fat have deleterious consequences on brain development, including decreased hippocampal size (Niculescu and Lupu, 2009). Importantly, studies revealed an increase in the inflammatory profile in obese/overweight pregnant women, particularly consistent is the increase of proinflammatory cytokine IL-6 and CRP (Pantham et al., 2015) and another recent meta-analysis revealed an increased risk for ASD conferred by overweight and obesity during pregnancy (Li et al., 2016). Another meta-analysis looking at population-based studies on the outcome of maternal weight in pregnancy also revealed increased risk of adult offspring to develop schizophrenia when the mother was obese or had a high BMI during early and late stages of pregnancy (Khandaker et al., 2012).

As with the genetic architecture, all of the individual components of nutritional imbalance can cumulatively contribute to a higher risk of neurodevelopmental disorders. A poor diet during pregancy generally lacks several of the micronutrients discussed as essential for neurodevelopment and for adequately regulating the immune system, as previously described in section “Specific Nutrient Imbalance and Inflammation,” while it can simultaneously include an excess of macronutrients, as is the case of “Western diets” that have an inflammatory effect or lead to inflammatory states like overweight and obesity.

### Potential Mechanism Behind Pathological Neurodevelopment

#### Genetic Programming in the Progeny

During development, the epigenome landscape is fully remodeled, therefore creating a time window during which adverse environmental exposure, including maternal diet imbalance, can trigger long-lasting changes on the actively differentiating cells of the offspring (Tarrade et al., 2015). This phenomenon occurring during prenatal and postnatal developmental stages is a process known as genetic programming of the cellular genome, transcriptome, or epigenome (Langley-Evans, 2009). Covalent modification of the chromatin as well as expression of microRNA can participate to fetal genetic or epigenetic programming, which can occur through adaptation mechanisms within the placenta or the developing fetus (Langley-Evans, 2009; Tarrade et al., 2015) (see Figure 1B).

The placenta directly contributes to intrauterine embryonic programming. Upon exposure to nutrient imbalance, the placenta adapts to help meet the needs of the growing and developing fetus. Adaptation and programming of the placenta involves alteration of the placental genome, transcriptome, and epigenome (Cox et al., 2015; Wilson et al., 2018) when exposed to various environmental factors, including inadequate maternal diet (Tarrade et al., 2015). Placental adaptation seems to occur in a sex-dependent manner (Rosenfeld, 2015). Male placentas are more dependent on the mother’s diet even if the nutrient transfer of their placentas is more efficient, because they possess lower storage capacities than female placentas (Eriksson et al., 2010). On the contrary, female placentas are more sensitive to the maternal environment leading to improved adaptation and lower burden on fetal development (Clifton et al., 2001; Rosenfeld, 2015). For instance, in a mHFD mouse model, placental DNA of female offspring becomes globally hypomethylated at “imprinted” genes involved in cellular, metabolic, and physiological functions (whose expression is determined by the parent and differently expressed depending on whether it was inherited from the mother or the father). In contrast, the placenta of male offspring showed lower methylation levels at steady-state. Together, these findings suggest placental adaptive capacities of offspring exposed to mHFD (Gallou-Kabani et al., 2010). Maternal diet can therefore modulate genetic programming of the placenta early on during development, and it can also influence the inflammatory profile of the placenta thus modifying its function through several mechanisms, rendering the progeny sensitive to neurodevelopmental alterations (Goeden et al., 2016).

In the offspring, maternal nutritional intake has directly been linked to modification of the epigenome landscape, i.e., expression of epigenetic regulators. Although research is expanding on the matter, limited knowledge remains. Nevertheless, a growing body of evidence has been accumulated on the link between maternal dietary consumption of vitamins or fats that highlight the genomic or epigenomic role of maternal nutrients in the offspring. Vitamin B, for instance, directly contributes to methionine synthesis (e.g., involved in DNA, polyamines, amino acids, phospholipids synthesis), which together with ATP forms a methyl group during the one carbon metabolic pathway. Low maternal levels of vitamins B can hence decrease methylation activity and directly contribute to the epigenetic remodeling of the progeny and alter genetic expression (Pepper and Black, 2011; Richmond and Joubert, 2017). Maternal vitamin D has also been associated with DNA methylation in the offspring germline and liver cells, which become hypomethylated in cases of maternal deficiency (Xue et al., 2016). However, these effects are subtle (Xue et al., 2016) and it remains plausible that epigenome-wide studies will reveal more drastic or important effects of vitamin D on the offspring’s epigenome signature.

More than the effect of specific nutrients, types of dietary patterns have also been suggested to modulate the offspring’s genetic programming. Mediterranean diet—enriched in fish, fruits, and vegetables, and with an increased intake of mono-unsaturated fatty acids (MUFAs)—decreased the risk of child maladaptive and atypical behaviors like ASD, anxiety, and depression in humans (House et al., 2018). This positive behavioral outcome of Mediterranean diet on the offspring was linked to methylation changes of imprinted regions *SEGC endonuclease/paternally expressed gene* (*PEG*) 10 in male and female offspring, as well as *maternally expressed gene* (*MEG*) *3* and *insulin-like growth factor 2* (*IGF2*) in male offspring (House et al., 2018). Dietary supplementation with PUFAs from algal source during pregnancy similarly increased *IGF2*-imprinted methylation (Lee et al., 2014). Other than imprinting on genes expressed paternally or maternally, fatty acids have been proposed to modulate the epigenome of genes involved in the biosynthesis of PUFAs like fatty acid desaturase 1/2 (Mennitti et al., 2015). Genome-wide studies on the blood of preadolescents with regard to their dietary intake demonstrated a significant correlation between methylation and total fat intake or ratio of MUFAs and PUFAs over total consumption of fats (Voisin et al., 2015). This suggests that in pregnant and nurturing women, fat intake and balance—including type of fats and proportions of other nutrients—may promote epigenetic changes within their progeny. Similarly, diet enriched in saturated and unsaturated fats has been linked to epigenetic changes in the offspring at the periphery (e.g., adipose tissue, heart, liver) (Attig et al., 2013; Keleher et al., 2018; Zhang et al., 2019) and in the CNS (Glendining and Jasoni, 2019). In the brain, mHFD animal model also demonstrated by ChIP-qPCR on the offspring hippocampus that mHFD leads to increased histone 3 K9 acetylation in males as well as decreased methylation of the histone K9 in females (Glendining and Jasoni, 2019). In another study by Grissom et al. (2015), mHFD was associated with the overexpression of epigenome modulator protein, DNA methyltransferase 1 (DNMT1), in the prefrontal cortex of the offspring. Moreover, mHFD mouse model investigating programming effect of mHFD on the offspring prior to pregnancy has demonstrated that hypertrophy and inflammation of adipose tissue, as well as overexpression of genes involved in fat deposition could only be prevented by long-term diet intervention prior to pregnancy (Summerfield et al., 2018), suggesting that offspring programming by the diet is a long-lasting mechanism. Strikingly, as we discussed in section “Carbohydrates and Fats,” imbalance of fatty acids as well as mHFD possesses inflammatory properties that seem to coincide with genetic reprogramming.

Limited information is available regarding the specific mechanisms of genetic programming, and how inflammation and epigenetic may be linked together; is the genetic reprogramming influenced by the inflammatory properties of the diet or are the two processes independent of each other? As genetic programming effects of maternal diet can partake in the development of neurodevelopmental disorders in the offspring, it is thus important to understand how inflammation and genes may interact together in the pathogenesis to comprehend how we could guide pregnant woman dietary consumption and limit their detrimental effects.

#### Neuroinflammation: Microglia and Blood Barrier

As previously discussed, nutrients can influence inflammatory processes in the mother, which in turn can promote inflammation in the progeny. Indeed, animal models of mHFD have described changes of inflammatory mediators (e.g., CD11b, IL-6, NFκB, TLR4) in the brain across the lifespan: neonate (Bilbo and Tsang, 2010; Graf et al., 2016), juvenile (Bilbo and Tsang, 2010), adolescent (Sasaki et al., 2014; Winther et al., 2018; Bordeleau et al., 2020), and adult (Bilbo and Tsang, 2010; Sasaki et al., 2013) stages. These changes in gene expression seem to occur differently between ages, sexes, and brain regions. Similarly, upon exposure to a maternal low *n*-3 PUFA diet, offspring’s brain transcriptomic signature revealed increased expression of transcript cluster associated to innate immune response and inflammation in *n*-3 PUFA-deficient mice (Madore et al., 2019). Moreover, both mHFD and maternal low *n*-3 PUFA studies reported changes in microglial density, morphology, mRNA/protein expression, or functions associated with brain development alterations, commonly observed in neuropsychiatric disorders (Bilbo and Tsang, 2010; Rey et al., 2018; Madore et al., 2019; Bordeleau et al., 2020). Although the impact of maternal nutrients other than fats and sugar has not been studied yet in the context of neuroinflammation changes in the offspring, it should be expected that their inflammatory effects in the mother have a ripple effect in the brain of the offspring, contributing to neurodevelopmental alterations. Moreover, microglia—the main immune cells of the CNS—are highly specialized in detecting and deploying inflammatory signals, which are especially sensitive to the inflammatory status during pregnancy that could modulate their physiological roles in brain development (Schwarz and Bilbo, 2012; Tay et al., 2018) (see Figure 1B).

Indeed, microglia are key immunocompetent cells that produce and respond to inflammatory cues. During neurodevelopment, these cues can influence microglial role and lead to changes in their modulation of neuronal network wiring and maturation (Tremblay et al., 2010; Paolicelli et al., 2011; Mallya et al., 2019), myelination (Bennett and Barres, 2017; Hagemeyer et al., 2017; Wlodarczyk et al., 2017), and of neurovascular development and maturation (Arnold and Betsholtz, 2013; Lacoste and Gu, 2015), throughout embryonic, fetal, juvenile, and adolescent neurodevelopment. Additionally, microglia undergo different stages of maturation during brain development, and this appears to be programmed *in utero* and dependent on maternal gut microbiota and inflammatory status of the mother (Matcovitch-Natan et al., 2016). Therefore, exposure to inflammatory-modulating environmental factors can modify microglial developmental roles and thus profoundly impact the offspring’s brain development.

Simultaneous to brain development, the offspring cerebrovascular system is developing and maturing from embryonic to adolescent stages (Arnold and Betsholtz, 2013). In the brain, several immune cues such as Notch, TNF-α, vascular endothelial growth factor (VEGF), and Wnt5a/11, released in part by microglia, contribute to neurovascular development and maturation (Arnold and Betsholtz, 2013; Lacoste and Gu, 2015). Peripheral and local inflammatory signals within the CNS can disturb its proper neurodevelopment, thus impacting neurovascular organization and function (Pearson-Leary et al., 2017; Van Dyken and Lacoste, 2018). In turn, this can impact on neuronal network function and plasticity (Sloan et al., 2010; Andreone et al., 2015; Lacoste and Gu, 2015) and render the offspring vulnerable to stress (Menard et al., 2017; Pearson-Leary et al., 2017). In fact, Menard et al. (2017) demonstrated that peripheral administration of cytokine IL-6 alone in steady-state was sufficient to induce neurovascular remodeling leading to increased permeability of the blood–brain barrier. Similarly, several other inflammatory mediators produced or modulated by maternal diet discussed above (see section “Specific Nutrient Imbalance and Inflammation”) could result in functional and organizational changes in the brain of the offspring (see Figure 1B). Although no intensive work has really been done on the matter, a leaky or dysfunctional blood–brain barrier could lead respectively to the recruitment of peripheral immune cells that could infiltrate and modulate the offspring’s neurodevelopment or to an inefficient transfer of adequate nutrients and energy to the CNS.

#### Gut Microbiome-Mediated Endotoxicity

Linked to its effects on inflammatory states and barrier permeability, HFD is well documented to have an important effect on the gut microbiome. In male mice, HFD alters gut microbiome within 4 weeks (Cani et al., 2008). Specifically, there are decreases for *Bifidobacterium* spp. and *Bacteroides*-related bacteria, as well as *Eubacterium rectale–Blautia coccoides* in mice (Cani, 2012), although a reduction in *Bacteroidetes* levels and an increase in *Firmicutes* and *Proteobacteria* were previously identified in the context of HFD, with or without inducing obesity (Hildebrandt et al., 2009; Ravussin et al., 2012). In addition, HFD can lead to metabolic endotoxemia (Cani et al., 2007), i.e., a sustained low-grade increase in circulating levels of LPS (a component of the outer membrane of Gram-negative bacteria), a lipoprotein acting as an inflammatory agent. While LPS has a physiological variation that peaks after feeding (Cani et al., 2008), persistent low-grade inflammation was observed after a mere 4 weeks of HFD consumption in adult mice (Cani et al., 2012).

The abnormal levels of LPS that infiltrate into the bloodstream after HFD appear to be due to a leaky gut–blood barrier. Indeed, when LPS levels were maintained abnormally high, there was a decrease in genetic expression of gut barrier tight-junction proteins zonula occludens-1 and occludin (Cani et al., 2012), but when treated with antibiotics, there was no disruption to the intestinal barrier, proposing therefore a role for gut microbiota in maintaining its integrity (Cani et al., 2008). Other forms of gut microbiota modulation through the use of pre- and probiotics have been shown to improve the integrity of the gut barrier and prevent metabolic endotoxemia in obese mice (Cani et al., 2009). In pregnancy, the issue of metabolic endotoxemia could be problematic for two main reasons: (1) it was associated with obesity, metabolic syndrome, and type 2 diabetes, all of which are pregnancy risk factors for the offspring, and (2) increased LPS in the bloodstream during pregnancy (even small but constant increases) amounts to an activation of the maternal immune system, which is known to have adverse consequences for the offspring.

Studies in pregnant women revealed that higher pregnancy BMI is linked to altered microbiome composition, with increase of bacteroides and staphylococcus, associated with excessive weight gain during pregnancy (Collado et al., 2008). In rats, bacterial population ratios change over the course of pregnancy and the alterations are exacerbated with the consumption of a HFD, regardless of whether it is accompanied by weight increase (Mann et al., 2017). Importantly, in a systematic review by Nelson et al. (2010), pre-pregnancy BMI was linearly associated to most adverse pregnancy outcomes and complications such as hypertension, preeclampsia and glucose intolerance/gestational diabetes. Not only is the greater BMI linked to metabolic alterations of fat and glucose pathways (Hellmuth et al., 2017), but obese women are observed to have increased circulating levels of the inflammatory mediator IL-6 (Ramsay et al., 2002; Basu et al., 2011) that appear to be linked to increased amounts of circulating endotoxins including LPS (Basu et al., 2011). Another group showed that overweight pregnant women had increased barrier permeability that was associated with higher blood concentrations of LPS (Mokkala et al., 2017). In addition, Laitinen et al. (2009) elegantly demonstrated in a cohort of normoglycemic pregnant woman that changes in glucose metabolism due to pregnancy could be improved by changing the gut microbiome *via* probiotics, with positive effects lasting past 12 months postpartum. This is congruent with experimental work showing that even in genetically obese mice (i.e., Ob/Ob and Db/Db mice), the use of prebiotics delayed and improved glycemic alterations in steady-state adulthood (Fernández de Cossío et al., 2017a; Song et al., 2019).

Together, the body of literature suggests that pregnancy itself produces microbiome, metabolic and peripheral immune changes in the mother that are similar to those observed in overweight and obese persons and which are associated to cardiovascular and glucose homeostasis issues *via* an augmented inflammatory status. The microbiome alterations are exaggerated further in the case of overweight or obesity before and during pregnancy. The gut microbiome can directly contribute to sustaining inflammatory processes in the mother, which in turn, can compromise offspring development, notably by acting on microglia (Castillo-Ruiz et al., 2018; Thion et al., 2018). Still, future investigation is indispensable to understand the molecular and cellular pathway linking maternal diet-gut microbiome to the offspring’s neurodevelopment.

### Perspectives and Concluding Remarks

In our day-to-day life, disadvantaged socioeconomic status as well as limited access to nutritious food are linked to the development of metabolic disorders associated with malnutrition, including overweight and obesity in pregnancy (Bleich et al., 2008; Martin et al., 2016). A growing body of evidence demonstrates the importance of an adequate quality and quantity of nutrients during pregnancy for the progeny’s development. As we discussed in this review, inadequate nutrient intake can create a systemic imbalance in the mother, compromising the developmental environment, thereby inducing an inflammatory state and/or a malabsorptive status. Under these conditions, the offspring might have an increased risk of developing neurodevelopmental disorders, which may be revealed with the occurrence of other environmental challenges later in life. As such, neurodevelopmental disorders have long been believed to mainly originate from genetic predisposition that upon exposure to certain environmental conditions lead to the expression of pathological behaviors. Maternal diet can exacerbate genetic vulnerability when it is deficient in essential nutrients or is grossly unbalanced; however, it can also directly influence fetal genetic programming and expression, and it has been shown to contribute to inflammation in both the mother and progeny with regulatory effects on brain development. As an influencing factor or part of the etiology, maternal diet is an actionable environmental contributor to brain pathology. Therefore, it may be a corner stone in setting the developmental outcomes of the progeny.

The tremendous importance of an optimal maternal diet for proper development and neurodevelopment encompassing cognitive and behavioral outcomes not only emphasizes the need to investigate thoroughly the long-term incidence of maternal lifestyle factors, namely dietary, but also stress exposure, meditation, physical exercise, etc. on the offspring. Moreover, the impact of the quality standard of food production (e.g., food-processing treatment, agriculture exposure, water and soil contamination) as well as water and air pollution should be considered. Epidemiological studies so far have also provided little information about food origin (e.g., GMO labels are not mandatory worldwide), as well as limited knowledge of environment contamination, pollution, use of pesticides, etc. In addition, little is known of how nutrition, metabolism, and inflammation are tied together, which highlights the need for longitudinal studies investigating their joint involvement in the progression, resolution, and modulation of offspring outcomes across life (infancy, childhood, adulthood, and aging).

In a similar way that women about to conceive or that are pregnant need to avoid toxic and infectious elements in the environment, it is also critical for them to pay particular attention to their nutritional intake. Indeed, environmental factors are actionable and nutrition can exert an effect on mechanisms that regulate and interact with genetic expression. If maternal diet can prime through its genetic and inflammatory effects the offspring to develop disorders in later life, then better understanding of dietary needs during fetal and neonatal development could lead us to understand the proper diet for a healthy progeny.

## Author Contributions

MB and LFC wrote the main manuscript of the review under the guidance and critical reviewing of MMC and M-ÈT. All the authors read, edited, and approved the final manuscript.

## Conflict of Interest

The authors declare that the research was conducted in the absence of any commercial or financial relationships that could be construed as a potential conflict of interest.
